# Efficacy of a new refractive multifocal contact lens for presbyopia

**DOI:** 10.1038/s41598-024-55918-5

**Published:** 2024-04-17

**Authors:** Do Young Kim, Hyunmin Ahn, Sukyung Lee, Ikhyun Jun, Kyoung Yul Seo, Sang Yeop Kim, Tae-Im Kim

**Affiliations:** 1 Department of Ophthalmology, Severance Hospital, Yonsei University College of Medicine, 50 Yonsei-ro, Seodaemun-gu, Seoul, 03722 Korea; 2 Siloam Eye Hospital, Seoul, Republic of Korea; 3 Eyejun Ophthalmic Clinic, Seoul, Republic of Korea; 4 Yonsei Plus Eye Center, Seongnam, Republic of Korea; 5 Clear Eye Clinic, Pyeongtaek, Republic of Korea

**Keywords:** Multifocal lens, Contact lens, Presbyopia, Clinical trials, Outcomes research, Quality of life

## Abstract

This prospective single-arm study aimed to evaluate the clinical efficacy and safety of a refractive multifocal contact lens for the correction of presbyopia in 22 patients. The participants underwent clinical examinations before and 1 week after wearing a refractive multifocal contact lens (OptaCon ZOOM). The primary endpoints were the corrected distance visual acuity (CDVA) and distance-corrected near visual acuity (DCNVA). Defocus curve, contrast sensitivity, and ocular surface disease index (OSDI) were analyzed. A slit-lamp examination was performed for safety analysis. Contact lens comfort and patient satisfaction were assessed using a questionnaire. No significant difference in CDVA was observed before and 1 week after refractive multifocal contact lens use (*p* = 0.127), whereas DCNVA was significantly improved after 1 week (*p* < 0.001). The contrast sensitivity was not significantly affected at any spatial frequency under mesopic or photopic conditions. OSDI was significantly increased (*p* = 0.023). The patient-reported satisfaction scores were 96.2, 91.9, and 85.0 out of 100 at far, intermediate, and near distances, respectively. No significant adverse events were observed. Refractive multifocal contact lenses improved near vision while maintaining distance vision in presbyopic patients, without compromising contrast sensitivity. The study results suggest that OptaCon ZOOM can be considered safe and effective for the correction of presbyopia.

## Introduction

Presbyopia is the age-related decrease in human accommodative amplitude that begins in the second decade of life and becomes clinically significant between 40 and 45 years of age^1,2^. Given the expected increase in life expectancy and proportion of aged population, presbyopia is a significant challenge for eye care practitioners due to its increasing prevalence with age^3^. The global prevalence of presbyopia is estimated to be 1.8 billion, or approximately 22% of the world’s population. Uncorrected or under-corrected presbyopia is estimated to be present in around 1.3 billion people and is significantly associated with reduced quality of life and decreased productivity in daily activities^4–6^.

Various methods including contact lenses, spectacles, and surgical interventions that modify the optics of the cornea or replace the crystalline lens have been proposed to correct the refractive error associated with presbyopia^4,7^. There has been constant interest in using contact lens for presbyopia because of their optical stability on the visual axis with the eye movement and cosmetic appeal^8^. Currently, various forms of multifocal contact lenses are available for presbyopia correction. The alternating-vision design has separate areas for near and distance vision. However, this design requires a translational change to relocate the most pupil to near zones and has the issue of image jumps, resulting in decreased depth perception and longer adaptation time^4,9^. In addition, simultaneous vision using multifocal contact lenses has been increasingly used for presbyopia correction. Simultaneous vision refers to foveal viewing of the images located at different distances along the optical axis for presbyopia correction. The pupil size and power distribution across the lens are essential for providing optical zones with different optical powers^1,10^. The concentric design features a central distance or near power zone surrounded by one or more annulus of opposite power in repeating pattern. The sharp transition between the near and distance zones can compromise the visual performance^4,11^.

The aspheric multifocal contact lens provides a gradual transition of the lens power between the distance and near powers, which increases the depth of focus by inducing spherical aberration. The induced spherical aberration may compromise the retinal image quality but it allows for increase in the vergence range at the same time^3^. Aspheric lens designs include center-near and center-distance designs. The center-near design has the highest plus power in the center, with a gradual decrease in power toward the periphery, whereas the center-distance design has the lowest plus power in the center, with a gradual increase in power toward the periphery^3,12,13^.

This study aimed to evaluate the efficacy of the new center-near aspheric refractive multifocal contact lenses that use simultaneous-image principle for the correction of presbyopia and report any side effects or complications after its application.

## Methods

### Ethics

This single-arm prospective study was performed in Severance Hospital, Yonsei University College of medicine, Seoul, Korea from June 2022 to August 2022. The study was approved by the Severance Hospital Institutional Review Board (IRB approval no. 2021-3385-001). It adhered to the principles of the Declaration of Helsinki. Informed consent was obtained from all subjects after the explanation of the nature and possible consequences of the study.

### Study participants

The current study was conducted on 24 patients with presbyopia aged 40 years and older. Two participants dropped out as they were lost to follow-up and 22 participants completed the study. The inclusion criteria were as follows: (1) presbyopia, addition of 0.75 D or more in both eyes; (2) astigmatism: up to − 1.00 D of cylinder power in both eyes (according to subjective refraction); (3) distance visual acuity of 0.3 logarithm of the minimal angle of resolution (logMAR) or better with contact lens fitting; and (4) no clinical history of ocular surface disease or dry eye symptoms. The exclusion criteria were as follows: (1) patients unsuitable for contact lens wear in their activities or occupations; (2) ocular disease or abnormalities that required regular topical medication or might have interfered with the ophthalmic examinations required for the current study; (3) the presence of chronic or moderate-to-severe ocular surface disease, including dry eye disease; and (4) a history of ocular surgery within 3 months. The dry eye symptoms were identified based on the report of the diagnostic methodology subcommittee of the international dry eye workshop (2007)^14^.

### Measurements

**Figure 1:**
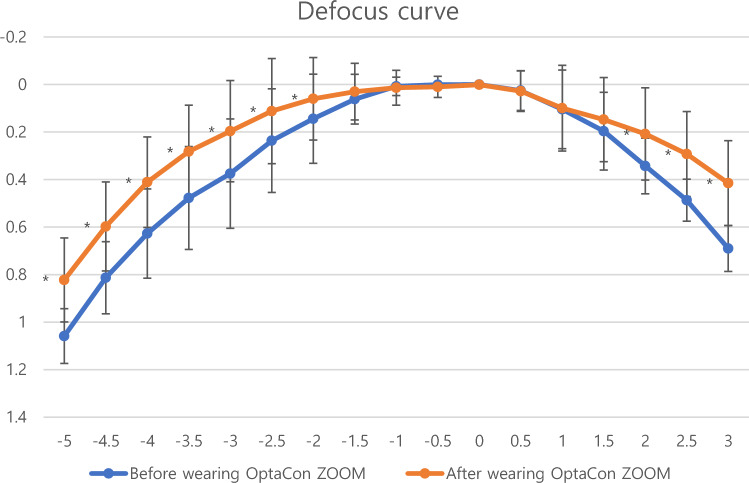
Defocus curve for the mean CDVA values before and after 1 week of multifocal soft contact lens wear. The curve had a U shape with a peak at 0.00D. The mean CDVA at the peak was not significantly different before and after 1 week of lens wear (*p*-value = 0.317). The result of direct comparison of defocus curve revealed that multifocal soft contact lens wear showed significant improvement of visual outcome for both near and far. It resulted in a significantly improved outcome for near vision particularly at − 2.00D to − 5.00D, corresponding with a 20–50-cm range (*p*-value < 0.05 in all cases). The result also showed significant improvement in outcome for far vision at + 2.00, + 2.50 and + 3.00D (*p*-value < 0.05 in all cases).

All participants underwent a complete ophthalmic examination prior to the study. Screening for ocular and systemic diseases, visual acuity, subjective refraction, near add power, eye dominance, and comprehensive slit lamp examination were performed. All the measurements were conducted by the same researcher (Tae-im, Kim). Keratometry was performed using a Topcon KR 8800 auto-kerato-refractometer (Topcon Corporation, Tokyo, Japan). The primary outcomes were corrected distance visual acuity (CDVA) and distance-corrected near visual acuity (DCNVA). Subjective distance refraction was performed using standard optometric measures and all measurements were taken with distance corrections. The VA measurement was cut at 0.00 logMAR. Distance visual acuity was measured at 4 m using an early treatment diabetic retinopathy study (ETDRS) logMAR chart (Precision Vision, La Salle, IL, USA) and near visual acuity was measured with the Logarithmic Visual Acuity Chart 2000 New ETDRS chart 1 for testing at 40 cm (Precision Vision, La Salle, IL, USA). Near add was defined as the minimum plus power over full subjective distance refraction required to read 0.0 logMAR print on the ETDRS chart. For defocus curve measurement, the eyes were defocused to + 3.00 D spherical from a subjective refraction value. Then, the trial lenses with negative spherical power were added in increments of -0.50 D until a power of -5.00 D was reached. The binocular visual acuity was measured using the ETDRS logMAR chart at 4 m with every increment. Defocus curves were obtained before and after 1 week of multifocal contact lens wear and were compared in each participant. We intended to measure each subject’s binocular visual acuity to simulate the subject’s visual performance at different distances and reflect the subjects’ experience in most day-to-day tasks. (Fig. 1) Binocular contrast sensitivity was measured under photopic (target luminance, 85 candelas/square meter [cd/m^2^]) and mesopic (target luminance, 3 cd/m^2^) conditions. The functional acuity contrast test (F.A.C.T) was performed using the Optec 6500 view-in test system (Stereo Optical Co., Inc., Chicago, IL, USA). Ocular surface disease index (OSDI) scores were obtained before and after 1 week of multifocal soft contact lens wear. All participants were asked to complete a questionnaire regarding visual symptoms, dry eye symptoms, overall satisfaction with the lenses, satisfaction with glasses, and recommendation to others. The questionnaire, created by combining and modifying previously reported questionnaire to evaluate postoperative symptoms, spectacle independence, and satisfaction, is shown in Table 1^5,15^. Slit lamp examination was performed to evaluate corneal erosion, edema, and peripheral injection for safety analysis.

**Table 1:** Questionnaire regarding discomfort, visual symptoms, and ability to perform tasks.

Question	Never (0)	Sometimes (1)	Half of the time (2)	Almost always (3)	Always (4)
1. Do you feel a sense of discomfort after wearing lenses?					
Photosensitivity					
Ocular pain					
Dryness					
Discomfort or Foreign sensation					
2. Do you experience any of the following visual symptoms after wearing lenses?					
Glare					
Halo					
Diplopia					
Fluctuation of visual acuity					
3. Do you have difficulty performing the following tasks?					
(a) Near distance tasks					
Reading medication instruction					
Reading newspaper					
Cleaning nails					
Using cellphone					
Reading magazine or books					
(b) Intermediate distance tasks					
Finding items in the kitchen					
Computer work					
Looking at the bathroom mirror					
Looking at the calendar					
Viewing a picture or a painting in a picture frame					
(c) Far distance tasks					
Reading road signs					
Identifying a person across the room					
Estimating the distance between cars					
Reading the house number or address					
Checking the time on the wall clock					

Question	Very dissatisfied (0)	Dissatisfied (1)	Neutral (2)	Satisfied (3)	Very satisfied(4)
4. Do you find wearing OptaCon ZOOM contact lens more satisfactory compared to wearing glasses?					
5. Would you recommend OptaCon ZOOM contact lens to other people?					

### Multifocal soft contact lens

**Figure 2:**
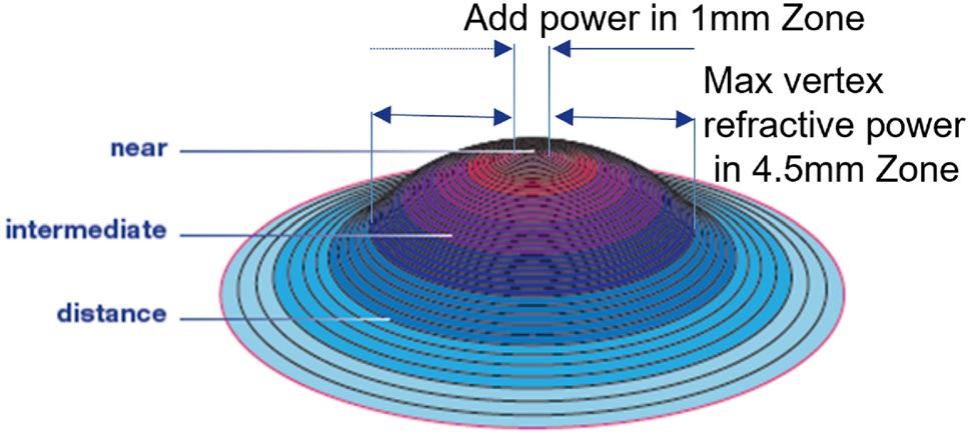
Diagram illustrating the lens design of OptaCon ZOOM. OptaCon ZOOM is manufactured from silicone hydrogel by cast molding method, with base curve of 8.6 mm and lens diameter of 14.2 mm. OptaCon ZOOM is designed with near vision optimized central 1 mm radius and gradual changes to negative spherical aberration in the periphery, with max vertex refractive power in 4.5 mm zone.

OptaCon ZOOM contact lens (INTEROJO Co. Ltd.. Pyeongtaek-si, Gyeonggi-do, Korea) is a disposable multifocal soft contact lens for presbyopia with an aspheric center-near design. (Fig. 2) The OptaCon ZOOM is designed with a 1 mm central radius optimized for near vision and exhibits gradual changes in power from the relative central plus to negative spherical aberration in the periphery. The OptaCon ZOOM is manufactured by cast molding with silicone hydrogel which is currently under a naming process. The OptaCon ZOOM has a base curve of 8.6 mm and a diameter of 14.2 mm. (Table 2) Lenses were prescribed for refractive error with a target of emmetropia, according to the manufacturer’s trial set guidelines. The same lens used for distance vision was placed in a trial frame for near vision assessment. Near visual acuity was assessed using binocular examination. Overcorrection was performed to achieve the optimal best-corrected visual acuity for both far and near distances. The final lens prescription was adjusted based on subjective refraction, as the OptaCon ZOOM is available in various add powers. Participants with add power lower than 1.0 D received a low addition lens of 1.0 D, those with add power between 1.0 D and 1.75 D received a moderate addition lens of + 1.50 D to + 2.0 D, and those with add power of + 2.0 D or higher received a high addition lens of 2.50 D. Participants were required to wear each daily disposable lens for at least 4 h daily for 1 week, including the follow-up visit. The lenses were discarded after a single day of wear. Lens removal was recommended in cases of severe adverse reactions including pain, abnormal discharge, and injection.

**Table 2:** The detailed parameter of OptaCon 1-day ZOOM Multifocal contact lens.

Parameter	Details
Base curve	8.6 mm
Diameter	14.2 mm
Power	+ 0.00D ~ − 6.00D (0.25D steps)
ADD power	Low(+ 1.00D), Med(+ 1.50D, + 2.00D), High(+ 2.50D)
Material	Silicone Hydrogel
Water content	45%
Manufacturing method	Cast molding
Oxygen permeability (Dk)	70 × 10^−11^ (cm^2^/s)[ml O_2_/(ml mmHg)
Oxygen transmissibility (Dk/t) @-3.00D	100 × 10^−9^ (cm/s) [ml O_2_/(ml mmHg)]

### Statistical analysis

A non-parametric test was used for statistical analysis because of the small sample size. Wilcoxon’s signed-rank test was used to compare the measurements obtained before and after OptaCon ZOOM wear. The statistical software package SPSS (version 22.0, IBM Corp., Armonk, NY, USA) was used for statistical analysis. A *p*-value of < 0.05 was considered statistically significant.

## Results

A total of 22 patients with presbyopia were included in the study. The majority were female (16 [72.72%]), and the mean age was 49.86 ± 4.23 years. The mean distance CDVA (DCDVA) was 0.003 ± 0.015 logMAR for the right eye and 0.00 ± 0.00 logMAR for the left eye. The mean DCNVA was 0.19 ± 0.15 logMAR for the right eye and 0.17 ± 0.15 for the left eye. The mean add power was 1.51 ± 0.49 D for both eyes. The mean spherical equivalent (SE) was − 1.98 ± 1.80 D for the right eye and − 1.85 ± 1.66 D for the left eye. Flat curvature power (K1), steep curvature power (K2), and steeper axis were 42.5 ± 2.08 D, 43.23 ± 2.29 D, and 89.86 ± 21.90° for the right eye and 42.32 ± 2.08 D, 43.03 ± 2.20 D, and 87.45 ± 25.60° for the left eye, respectively. (Table 3).

**Table 3:** Patient demographics.

Baseline characteristics	OD	OS
Visual acuity		
DCDVA (log MAR)	0.00 ± 0.015	0.00 ± 0
DCNVA (log MAR)	0.19 ± 0.15	0.17 ± 0.15
Add power (D)	1.51 ± 0.49	1.51 ± 0.49
Refractive errors		
Sph (D)	− 1.78 ± 1.80	− 1.68 ± 1.69
Cyl (D)	− 0.40 ± 0.35	− 0.34 ± 0.26
Axis (°)	116 ± 63	101 ± 59
K values		
K1 (D)	42.50 ± 2.08	42.32 ± 2.08
K2 (D)	43.23 ± 2.29	43.03 ± 2.20
Axis (°)	90 ± 22	87 ± 26

Values are presented as mean ± standard deviation.

*OD*—right eye, *OS*—left eye, *DCDVA*—distance corrected distance visual acuity, *DCNVA*—distance corrected near visual acuity, *OSDI*—ocular surface disease index, *Sph*—spherical power of subjective refraction, *CYL*—cylindrical power of subjective refraction.

The DCDVA was not significantly different before and after 1 week of contact lens use (0.00 ± 0.11 vs. 0.01 ± 0.03, *p* = 0.127). DCNVA showed significant improvement from 0.19 ± 0.15 logMAR to 0.04 ± 0.08 logMAR after 1 week of contact lens wear (*p* < 0.001). (Table 4).

**Table 4:** Visual acuity of enrolled eye with and without wearing multifocal contact lenses.

	Before wearing lens	After wearing lens	*P* value
DCDVA (logMAR)	0.00 ± 0.11	0.01 ± 0.03	0.13
DCNVA (logMAR)	0.19 ± 0.15	0.04 ± 0.08	< 0.001
OSDI	16.15	28.45	0.02

Values are presented as mean ± standard deviation.

*DCDVA*—distance corrected distance visual acuity, *DCNVA*—distance corrected near visual acuity, *CNVA*—corrected near visual acuity, *OSDI*—ocular surface disease index.

**Figure 3:**
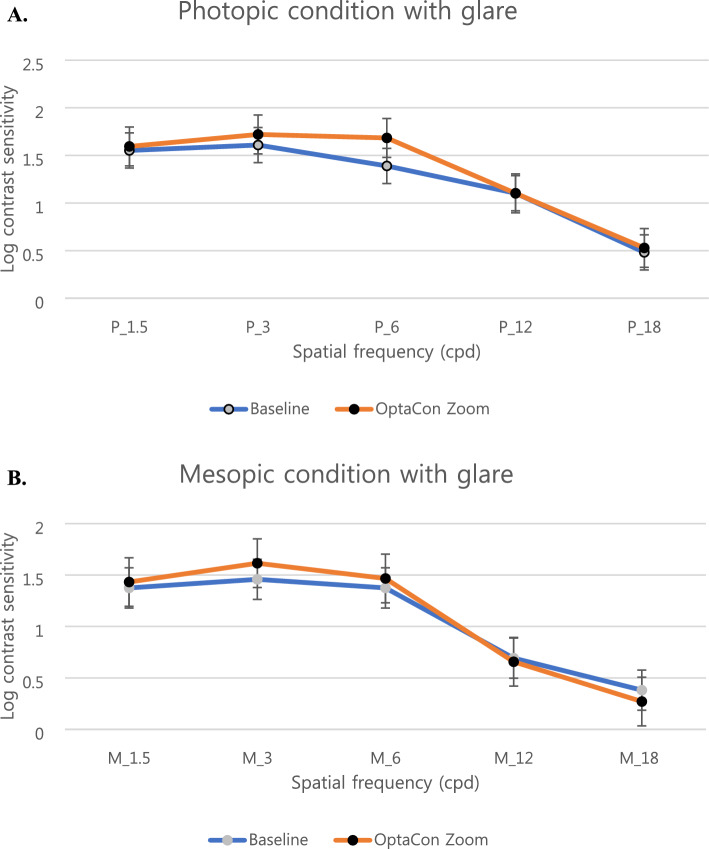
Contrast sensitivity values before and after 1 week of OptaCon ZOOM wear for the correction of presbyopia. The images show binocular contrast sensitivity values under photopic (**A**) and mesopic (**B**) conditions.

There was no significant difference in the results of the binocular contrast sensitivity test for any frequency under photopic and mesopic conditions (Fig. 3). Thus, multifocal soft contact lens wear was not significantly associated with a decrease in binocular contrast sensitivity under mesopic or photopic conditions.

The OSDI score was significantly increased after 1 week of multifocal soft contact lens use (*p* = 0.02; Table 4). No significant adverse events were observed on slit lamp examination. Dryness was the most common discomfort symptom with the mean score of 1.38 ± 1.24 out of 4, followed by foreign sensation with the mean score of 1.29 ± 1.27 out of 4. For visual symptom, diplopia and fluctuation of visual acuity were the most common symptoms with the mean score of 1.81 ± 1.24 and 1.57 ± 1.21 out of 4, respectively. The mean patient-reported satisfactory scores converted to a score of 100 obtained by the questionnaire were 96.2, 91.9, and 85.0 for distance, intermediate, and near vision, respectively. The lens satisfaction score comparing glasses and the score for recommending the product were slightly above average, with scores of 2.10 and 2.05 out of 4, respectively. (Table 5).

**Table 5:** Questionnaire regarding discomfort, visual symptoms, and ability to perform tasks.

	Mean ± SD
1. Do you feel a sense of discomfort after wearing lenses?	
Photosensitivity	0.52 ± 0.68
Ocular pain	0.43 ± 0.68
Dryness	1.38 ± 1.24
Discomfort or Foreign sensation	1.29 ± 1.27
2. Do you experience any of the following visual symptoms after wearing lenses?	
Glare	0.67 ± 0.86
Halo	1.19 ± 1.21
Diplopia	1.81 ± 1.24
Fluctuation of visual acuity	1.57 ± 1.21
3. Do you have difficulty performing the following tasks?	
(a) Near distance	
Reading medication instruction	3.05 ± 1.02
Reading newspaper	3.43 ± 0.93
Cleaning nails	3.33 ± 1.15
Using cellphone	3.33 ± 0.87
Reading magazine or books	4.00 ± 0.97
(b) Intermediate distance	
Finding items in the kitchen	3.14 ± 0.00
Computer work	3.71 ± 1.01
Looking at the bathroom mirror	3.71 ± 0.72
Looking at the calendar	3.62 ± 0.80
Viewing a picture or a painting in a picture frame	3.90 ± 0.44
(c) Far distance	
Finding items in the kitchen	3.90 ± 0.44
Computer work	3.90 ± 0.44
Looking at the bathroom mirror	3.90 ± 0.45
Looking at the calendar	3.71 ± 0.72
Viewing a picture or a painting in a picture frame	3.81 ± 0.60
4. Do you find wearing OptaCon ZOOM contact lens more satisfactory compared to wearing glasses?	2.10 ± 1.04
5. Would you recommend OptaCon ZOOM contact lens to other people?	2.05 ± 0.80

## Discussion

Presbyopia is expected to increase sharply with the rapid growth of the aging population; therefore, effective treatment modalities and strategies for the correction of presbyopia are becoming increasingly important (2). The present study analyzed the clinical performance of a refractive multifocal soft contact lens for the correction of presbyopia and found that the lens improved near vision while correcting distance vision. Previous studies on similar age groups that assessed the visual performance of presbyopic contact lens showed similar results^16,17^.

Monovision, which is a system for fitting one eye for distance vision and the other for near vision, is another common option for contact lens correction of presbyopia. In a previous randomized controlled study, both multifocal and monovision contact lens wearers maintained at least 20/20 binocular vision, whereas multifocal wearers showed significantly lower vision at near distances under low-contrast conditions. However, self-reported quality of life and lens preference were significantly higher with multifocal contact lenses than in those with the baseline and monovision, possibly because of comparable visual acuity without compromising stereoacuity to the same degree as in monovision^12^. Monovision performs better for distance and near visual acuity, whereas a center-near aspheric multifocal contact lens performs better for stereoacuity and near-range clear vision. This implies that center-near aspheric simultaneous-vision multifocal lenses provide a better balance of real-world function^18^. Another previous study showed that both near and distance visual acuity were significantly improved in patients with presbyopia with multifocal contact lenses compared to uncorrected baseline visual acuity, with minor adverse effects^7^. Our study result showed significant improvement in DCNVA to fair value of 0.04 ± 0.08 logMAR. For the result of other MCLs, near VA was reported to be 0.05 ± 0.06 for PureVision multifocal, 0.49 ± 0.21 in mild presbyopic group and 0.48 ± 0.20 in moderate to severe presbyopia group for Air Optix® Aqua Multifocal at 40 cm, and 0.102 ± 0.055 for Morning QI-plus^1,19,20^.

The contrast sensitivity test is associated with the performance of spatial vision in both size and contrast, and it correlates better with identifying real-world objects, providing a better measure of visual sensitivity and quality than visual acuity alone^18,21^. Our study showed no significant difference in contrast sensitivity before and after multifocal contact lens wear at all spatial frequencies under both mesopic and photopic conditions. The results of the current study correlate with those of previous studies comparing different types of lenses^18,22^. However, some studies have reported conflicting results regarding the impact of multifocal contact lenses on contrast sensitivity, with a greater reduction in contrast sensitivity shown with multifocal lens wear than with single vision lenses under low-light conditions^10,23,24^.

In our study, the OSDI was significantly increased after wearing contact lenses compared with the baseline evaluation. These results appear to be consistent with those of other studies, in which contact lenses seemed to disrupt the ocular surface and tear film^25,26^. Given the high prevalence of dry eye disease in older population, dry eye symptoms must be considered when prescribing contact lens for presbyopia correction. However, the higher proportion of females in the current study may have led to an increase in the OSDI score, since previous studies have reported that females are more likely to report symptoms of dryness regardless of age^26,27^. Moreover, the apparent lack of association between symptoms of dry eye and available clinical tests has been reported in previous studies^26,28,29^.

Although the multifocal refractive contact lens significantly improved near vision, patient satisfaction was limited by the lack of near-full correction expected by the patient. The OptaCon ZOOM has a near power zone of 1.0 mm, which may result in the symptoms of diplopia and fluctuation of vision and relatively narrow depth of focus for intermediate distances because of the sharp dioptric transition from near center zone to peripheral optic zone. Further studies are required to address this issue.

The current study had some limitations. First, the study had a small sample size and a short follow-up period of 1 week after lens wear. However, we expected relatively short adaptation period to be required since applying contact lenses provides an immediate improvement in VA. We expected a follow-up period of 1 week would be sufficient as it was in past studies. Still, the long-term efficacy of multifocal soft contact lenses should be further investigated in future studies with a larger sample size and a longer follow-up period. Furthermore, all patients included in this study had either no prior experience with contact lenses or had not used them for an extended period. The difficulty that the participants faced in adapting to the lenses may have influenced the results of the trial, including visual symptoms, ocular discomfort, and overall satisfaction. Taking this aspect into account during the prescription process may lead to higher levels of satisfaction.

In conclusion, refractive multifocal contact lenses improved both far and near vision in patients with presbyopia without compromising contrast sensitivity. The results of the current study suggest that an aspheric center-near designed multifocal contact lens can be considered a minimally invasive measure for presbyopia correction.

## Data Availability

All data generated or analyzed during this study are included in this published article. The datasets used during the current study are available from the corresponding author on reasonable request.
